# Supplementary material for: The Construction and Exploration of a Comprehensive MicroRNA Centered Regulatory Network in Foxtail Millet (Setaria italica L.)
Source: Front Plant Sci. 2022 May 6;13:848474. doi: 10.3389/fpls.2022.848474 (PMC9121102; doi:10.3389/fpls.2022.848474)
Supplement: Supplementary file 1 [file Data_Sheet_1.zip › Sit-miR2275a_Seita.8G177500.1_99_TPlot.pdf]

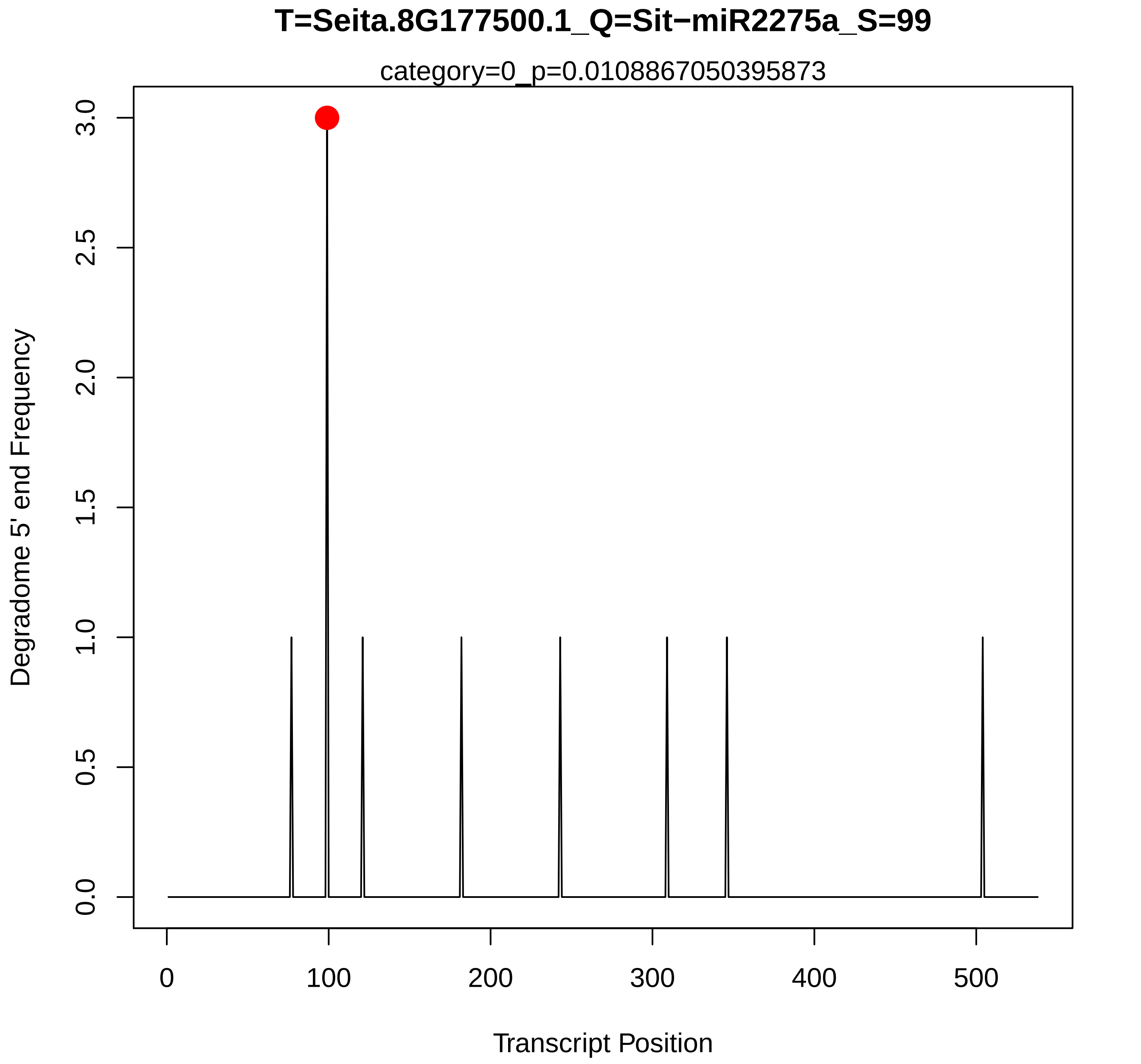
T=Seita.8G177500.1_Q=Sit−miR2275a_S=99
category=0_p=0.0108867050395873
Degradome 5' end Frequency
3.0
2.5
2.0
1.5
1.0
0.5
0.0
0
100
200
300
400
500
Transcript Position
